# A panoply of neoplastic plasma cells

**DOI:** 10.1002/jha2.1010

**Published:** 2024-10-24

**Authors:** Radu Chiriac, Sophie Gazzo

**Affiliations:** ^1^ Laboratoire d'hématologie biologique Hospices Civils de Lyon, Centre Hospitalier Lyon Sud Pierre‐Benite France

**Keywords:** anaplastic, myeloma, plasma cell

1

A 70‐year‐old man with a 5‐year history of prostate adenocarcinoma, currently in complete remission, presented with pancytopenia and pain in his lower ribs. No abnormal circulating cells were observed.

The positron emission tomography‐computed tomography (PET‐CT) scan revealed fractures of the two last right ribs and abnormal F‐18 fluorodeoxyglucose (^18^F‐FDG) uptake in the axial skeleton. A bone marrow aspirate revealed the presence of abnormal plasma cells, varying from small to medium‐sized mononucleated forms to bi‐, tri‐, quadri‐, and even pentanucleated forms, resembling anaplastic plasma cells (Figure 1, Panel A, May‐Grunwald Giemsa stain, x100 objective). Flow cytometry confirmed a lambda‐restricted population of CD38+/CD138+ plasma cells with loss of CD45, CD19, CD56, and CD117. Blood work showed immunoglobulin G lambda paraprotein. Metastatic adenocarcinoma was excluded by immunohistochemistry. Interphase fluorescence in situ hybridization (FISH) analysis revealed a del(17)(p13.1) involving *TP53* (Figure 1, Panel B, TP53/NF1 deletion probe) in 95% of cells, including both diploid and tetraploid clones, and an IgH(14q32) abnormality (Figure 1, Panel C, diminished FISH signals, IGH+ break‐apart probe). No t(4;14)(p16;q32) FGFR3/IGH translocation was detected. These findings were consistent with multiple myeloma displaying anaplastic features.

**FIGURE 1:**
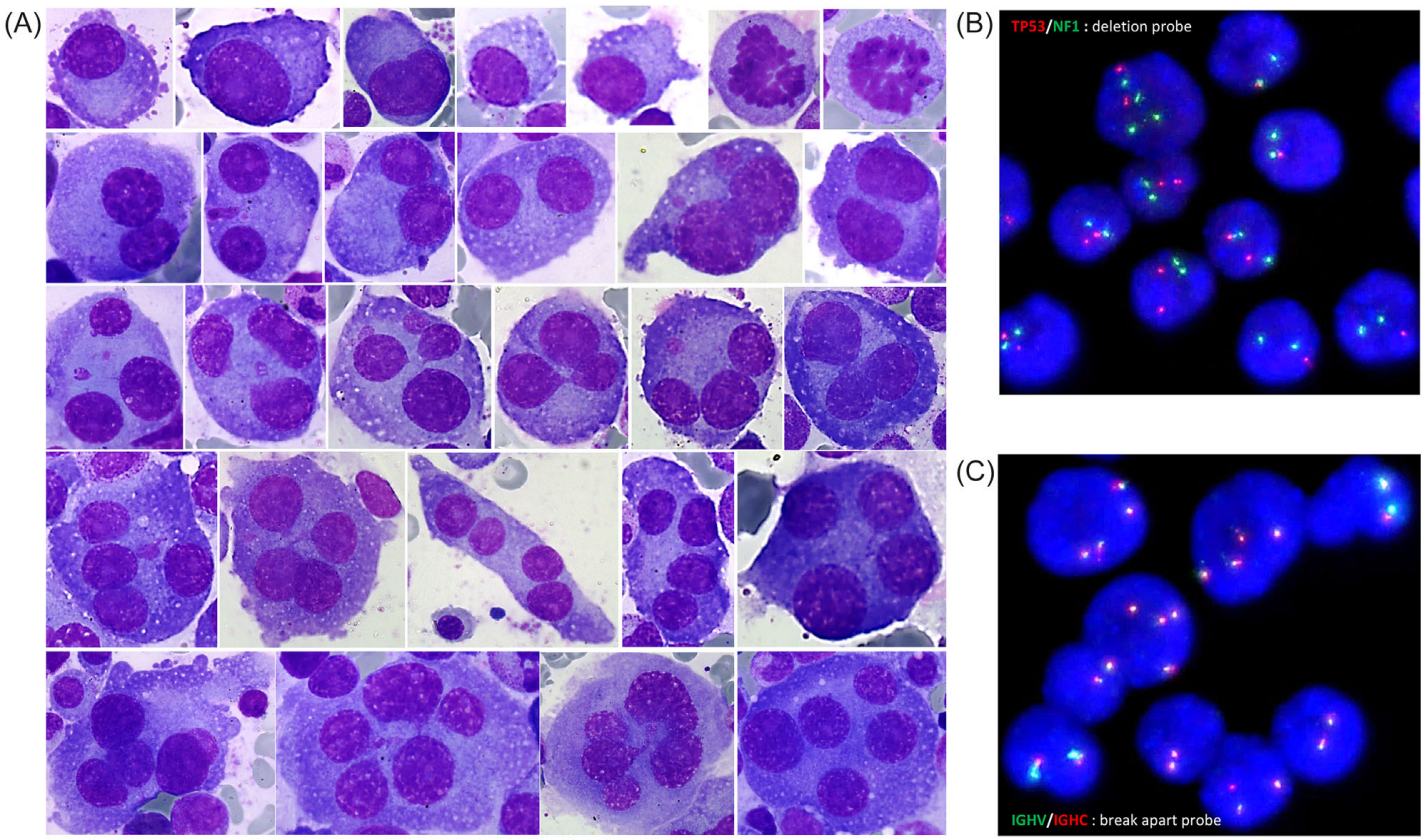
Panel A: May‐Grunwald Giemsa stain, x100 objective, showing morphological changes in anaplastic myeloma cells. Panel B: fluorescence in situ hybridization (FISH) with a TP53/NF1 deletion probe. Panel C: FISH with an IGH break‐apart probe, showing diminished signals.

The patient, ineligible for a transplant, was started on bortezomib, cyclophosphamide, and dexamethasone. After completing four cycles with a very good partial response, he was admitted to the hospital with worsening respiratory distress and grade 4 neutropenia. A chest CT scan revealed hazy ground‐glass opacities scattered throughout both lungs, with denser abnormalities in the lower lobes bilaterally. There were no other signs or symptoms suggestive of pneumonia. The patient was treated with high‐dose methylprednisolone and noninvasive positive pressure ventilation for bortezomib‐induced pneumonitis but showed no improvement. Unfortunately, two weeks after admission, the patient passed away due to ventilator‐associated pneumonia.

This case highlights that while most plasma cell neoplasms are recognizable by classic morphology, some variants with unusual features can mimic anaplastic carcinoma or lymphoma. Their pleomorphic multinucleated morphology can resemble that of multinucleated carcinomas or even dysplastic megakaryocytes due to their multilobed nuclei and, abnormal nuclear distribution, complicating the differentiation from metastatic carcinoma, myelodysplastic syndrome, or plasmablastic lymphoma [1]. Additionally, the patient's prior history of adenocarcinoma further challenged the diagnostic process in this case.

## AUTHOR CONTRIBUTIONS

Radu Chiriac and Sophie Gazzo wrote the manuscript, conducted the cytological studies, and performed the cytogenetic studies. All authors contributed to the final manuscript.

## CONFLICT OF INTEREST STATEMENT

The authors declare no conflicts of interest.

## FUNDING INFORMATION

The authors received no specific funding for this work.

## ETHICS STATEMENT

This manuscript respects the ethics policy of CHU Lyon for the treatment of human research participants.

## PATIENT CONSENT STATEMENT

No patient‐identifying data were used. The authors did not obtain written informed consent from the patient but the patient did not object to his data being used for research purposes (as required by the ethics policy of CHU Lyon).

## CLINICAL TRIAL REGISTRATION

The authors have confirmed clinical trial registration is not needed for this submission.

## Data Availability

Data sharing is not applicable to this article as no new data were created or analyzed in this study.
